# Supplementary material for: The effect of tides on nearshore environmental DNA
Source: PeerJ. 2018 Mar 19;6:e4521. doi: 10.7717/peerj.4521 (PMC5863721; doi:10.7717/peerj.4521)
Supplement: Supplemental Information 1 [file peerj-06-4521-s001.pdf]

Supplemental Information: Kelly et al. Tides and eDNA

Supplemental Table 1: Sampling Sites in Hood Canal, Washington, USA. Samples were taken intertidally, in water less than 1m deep.

Site	Latitude	Longitide
Lilliwaup State Park	47.46	-123.1
Potlatch State Park	47.38	-123.2
Twanoh State Park	47.38	-123.0

Supplemental Table 2: Apportioning variance in Jaccard distance among eDNA communities due to Tide, Site, Sampling Event, Sampling Bottle, and PCR Reaction (Residuals).

	Df	SumsOfSqs	MeanSqs	F.Model	R2	P.value
Tide	1	0.6607	0.6607	2.521	0.0218	0.001
Site	2	2.9267	1.4633	5.583	0.0967	0.001
Sampling Event	8	4.4366	0.5546	2.116	0.1466	0.001
Sampling Bottle	23	7.0322	0.3057	1.167	0.2324	0.001
Residuals	58	15.2016	0.2621	NA	0.5024	NA
Total	92	30.2577	NA	NA	1.0000	NA

Supplemental Table 3: Summary of unique annotations, by taxonomic rank, in the COI dataset.

Phylum	Classes	Orders	Families	OtherRank
Proteobacteria	4	4	2	0
Bacillariophyta	11	17	24	5
Dinoflagellata	7	12	12	0
Ascomycota	5	7	10	6
Basidiomycota	2	3	3	2
Mucoromycota	1	1	1	0
Phaeophyceae	6	14	37	4
Annelida	5	9	12	4
Arthropoda	20	67	80	43
Brachiopoda	0	0	1	0
Bryozoa	1	1	1	0
Chordata	13	16	17	3
Cnidaria	5	13	18	4
Echinodermata	1	1	2	0
Gastrotricha	1	1	1	0
Mollusca	7	20	23	3
Nematoda	1	1	1	0
Nemertea	1	2	2	2
Porifera	7	7	7	1
Rotifera	1	1	1	1
Rhodophyta	5	13	15	0
Chlorophyta	5	7	11	3
Streptophyta	4	4	4	0

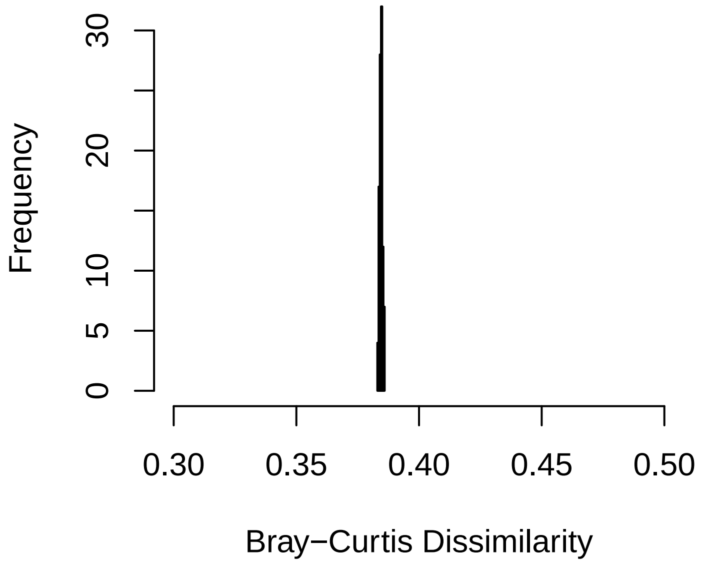

Supplemental Figure 1: Differences among 100 rarefaction draws from the overall dataset, expressed as median Bray-Curtis dissimilarities. The lack of variation in median dissimilarity among rarefaction draws underscores the fact that the results we present in the main manuscript do not differ substantially among different draws.

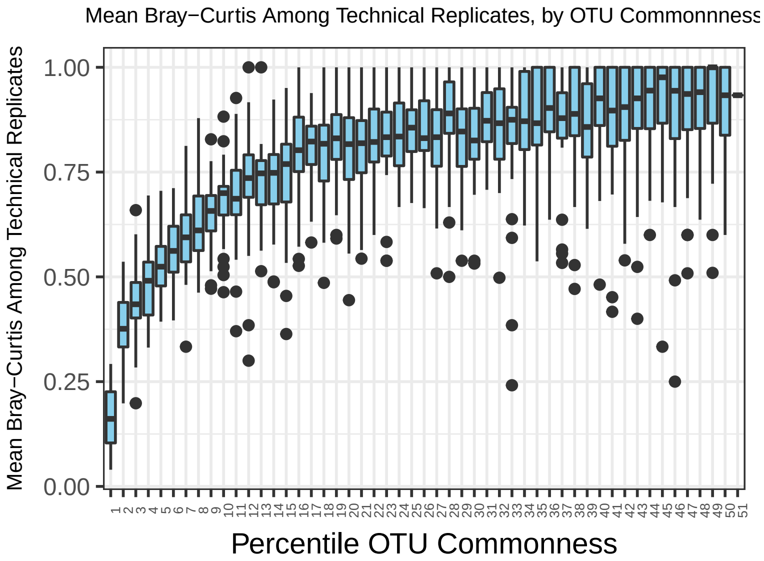

Supplemental Figure 2: Variation among technical (PCR) replicates, expressed as Bray-Curtis dissimilarity among replicates using data subsets according to OTU commonness. Replicates are similar with respect to common OTUs, but stochasticity quickly dominates as OTUs become rarer, such that PCR replicates appear quite different with respect to OTUs in the bottom 90 percent of commonness.

Supplemental Table 4: Coordinates for Washington Department of Ecology water quality sampling sites, which encompass the waters we sampled for the study presented in the main text.

Site Name	Latitude	Longitude
Admiralty Inlet	48.03	-122.6167
Send Creek	47.667	-122.82
Hamma Hamma	47.5383	-123.0083
Great Bend	47.3567	-123.0233
Lynch Cove	47.3983	-122.9283

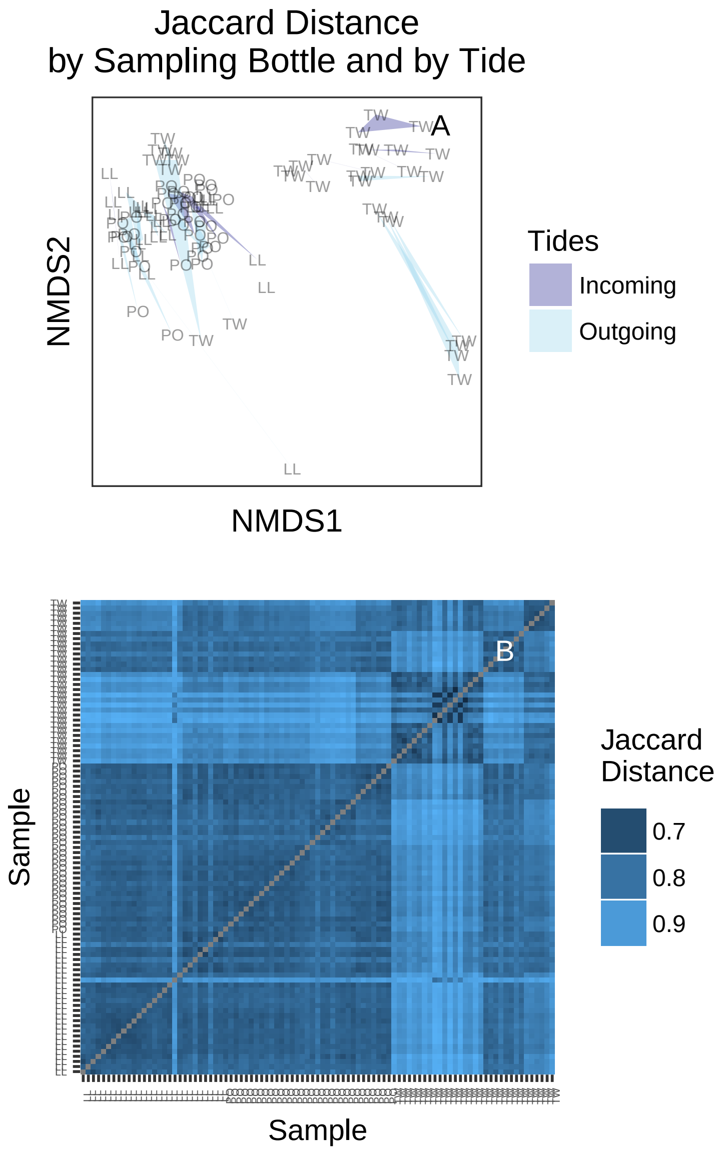

Supplemental Figure 3: (A) Ordination plot (non-metric multidimensional scaling; NMDS) plot of Jaccard distances among sequenced replicates, by sampling bottle (polygon) and tide (polygon color). Polygons connect communities sequenced from replicate PCR reactions of the same sampled bottle of water. (B) The same data shown as a heatmap, ordered by site identity. Only the Twanoh samples (upper right) stand out as having substantial heterogeneity, reflecting the two different communities present during different sampling events at that site. Site labels: TW = Twanoh, PO = Potlatch, LL = Lilliwaup. Note that Jaccard upweights rare (and therefore stochastic) OTUs relative to the same analyses presented in the main text with Bray-Curtis dissimilarities, and accordingly the Jaccard data have a high level of noise.

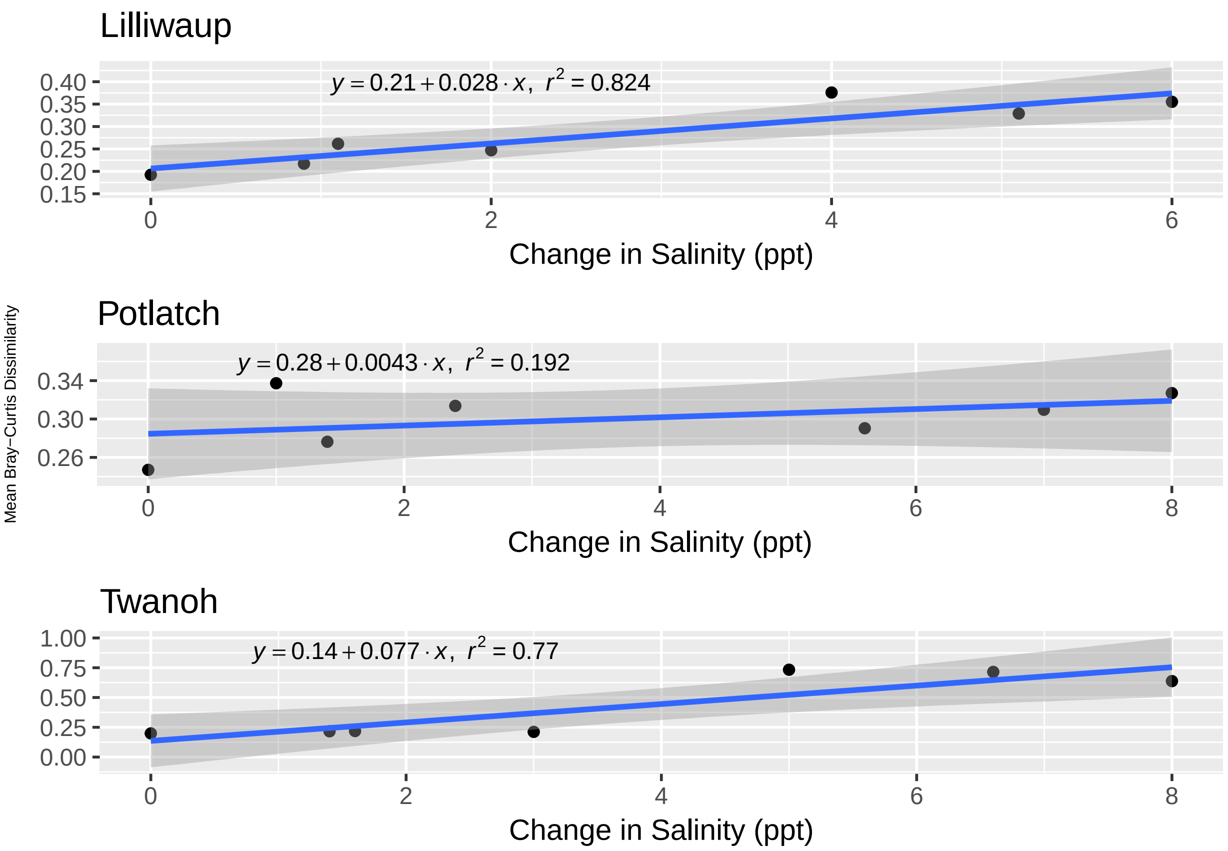

Supplemental Figure 4: Changes in eDNA community are associated with changes in salinity at each site; note the different scales in both the x and y axes.

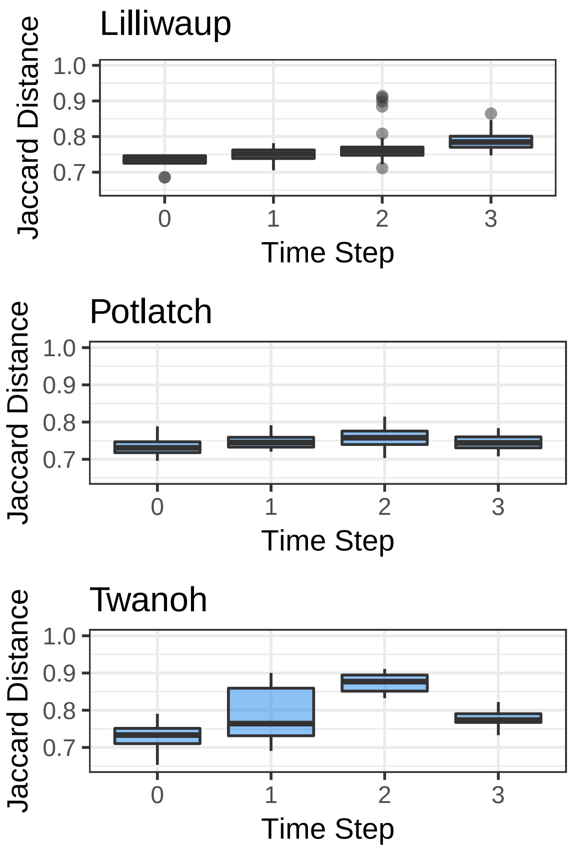

Supplemental Figure 5: Comparison of Jaccard distances within a reference sampling event (Time step = 0) and between the reference sample and subsequent samples at the same site (Time Steps 1, 2, and 3). Subsequent time steps reflect the accumulation of ecological eDNA differences over hours as the tide moves in and out. Sites shown individually.

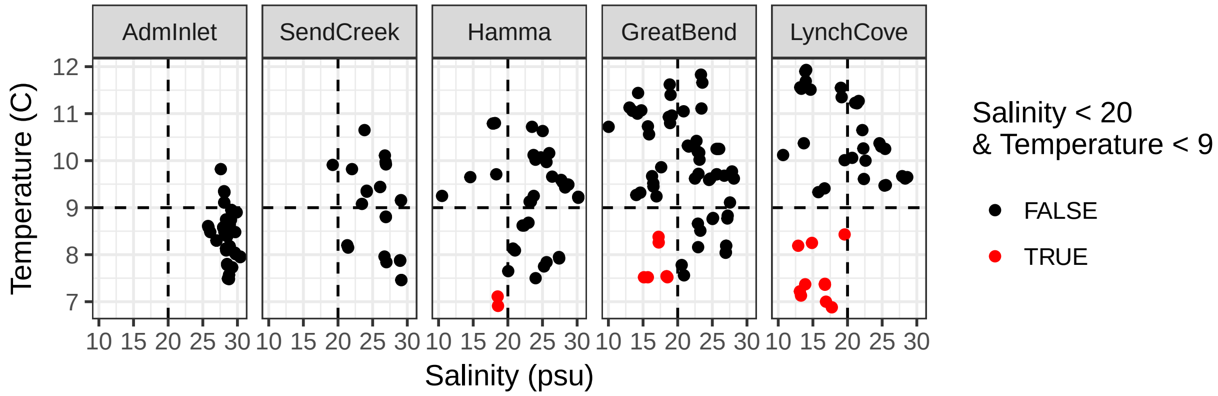

Supplemental Figure 6: Contextual water data for the month of March from the Washington State Department of Ecology (https://fortress.wa.gov/ecy/eap/marinewq/mwdataset.asp). Plots are arranged north to south, with the southernmost point being Lynch Cove, near our sampled site of Twanoh. Red points indicate Temperature/Salinity data in the range of those in which we observed eDNA Community 2; these are far more common in the southern end of Hood Canal than in the north, which has a stronger oceanic influence. The data show more southern points in the Hood Canal have an increased likelihood of cold, fresh water in March (red points). These make up the following proportions: Admiralty Inlet = 0, Send Creek = 0, Hamma Hamma =0.05, Great Bend = 0.11, Lynch Cove = 0.28.

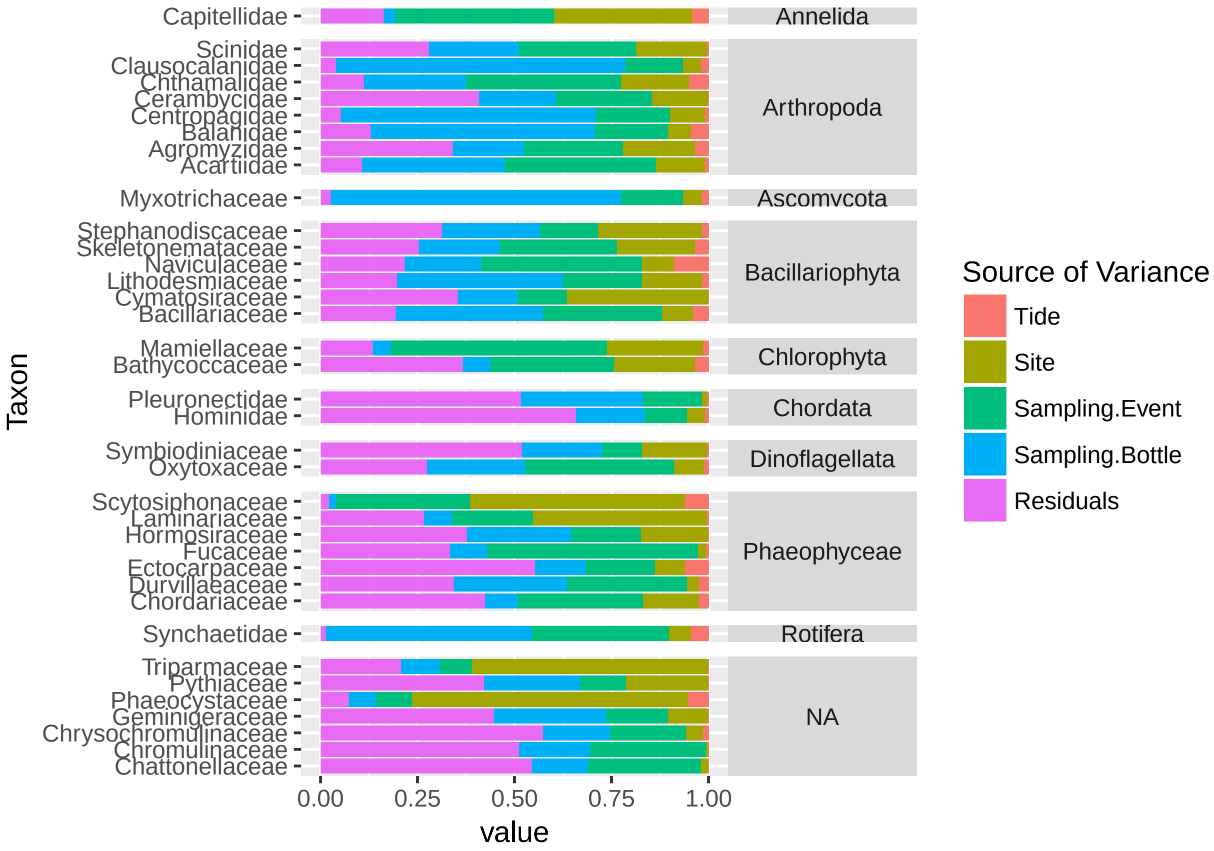

Supplemental Figure 7: The portion of variance in community distances due to tide, sampling site, sampling event, sampling bottle, and PCR reaction (residual variance) broken down by taxonomic Family (left-hand axis labels) and phylum (right-hand axis labels). Those Families with phyla given as 'NA' have unsettled higher taxonomy and therefore have no assigned phylum according to the NCBI database. Note that here, distances are calculated as Euclidian rather than Bray-Curtis, in order to accommodate the many instances in which zero reads from a particular Family where detected at a given site. Families with at least 100 reads in the overall dataset shown.

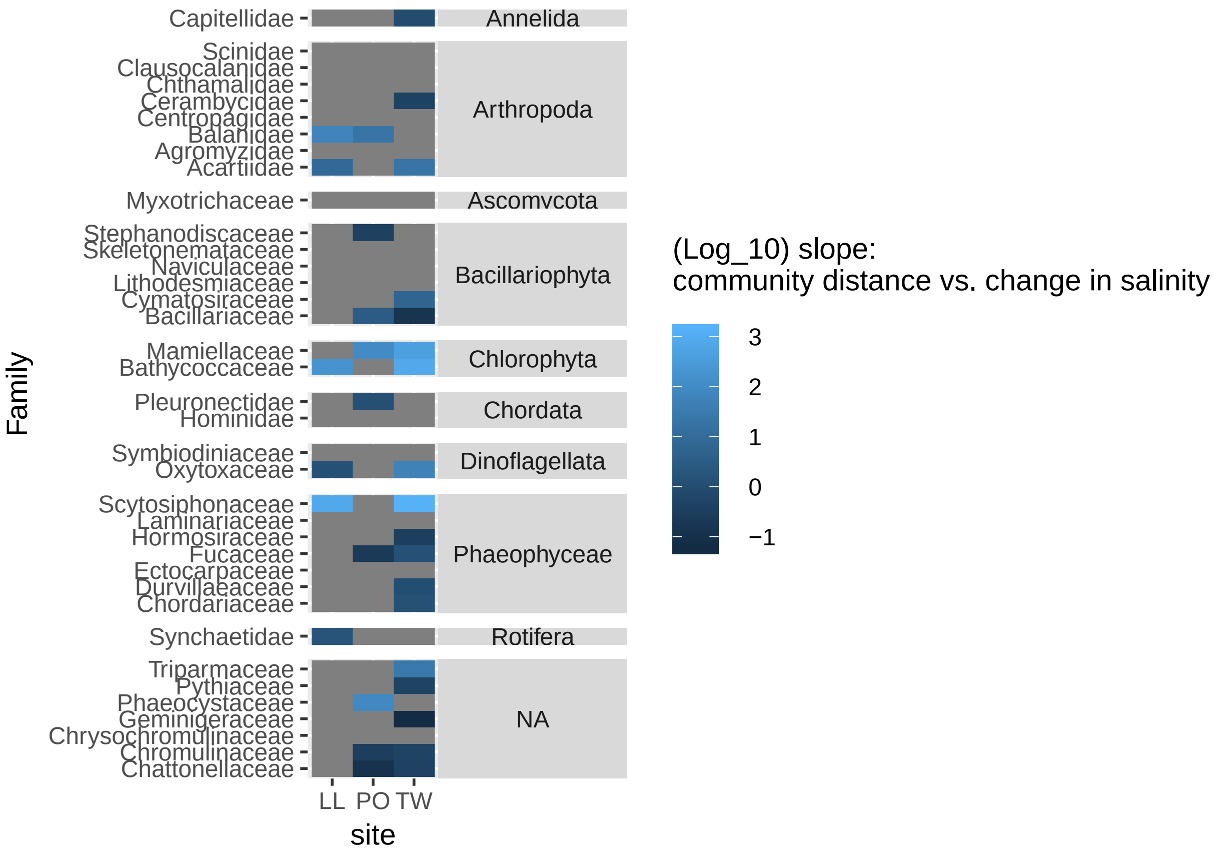

Supplemental Figure 8: Family-level community distances associated with changes in water mass (as measured by change in salinity), broken down by sampling site. Statistically significant slopes (alpha = 0.05) shown; base-10 logs plotted for legibility. Note that here, community distances are calculated as Euclidian rather than Bray-Curtis, both to accommodate the many instances in which zero reads from a particular Family where detected at a given site and to appropriately calculate salinity differences. Lighter-colored boxes indicate stronger associations between community identity (within Family) and salinity. These instances of strong association occur across phyla and life-histories, for example in benthic metazoans and multicellular algae (Balanidae and Scytosiphonaceae) as well as in single-celled planktonic groups such as the Chlorophyta.
